# A Method Based on Artificial Intelligence To Fully Automatize The Evaluation of Bovine Blastocyst Images

**DOI:** 10.1038/s41598-017-08104-9

**Published:** 2017-08-09

**Authors:** José Celso Rocha, Felipe José Passalia, Felipe Delestro Matos, Maria Beatriz Takahashi, Diego de Souza Ciniciato, Marc Peter Maserati, Mayra Fernanda Alves, Tamie Guibu de Almeida, Bruna Lopes Cardoso, Andrea Cristina Basso, Marcelo Fábio Gouveia Nogueira

**Affiliations:** 1 Universidade Estadual Paulista (Unesp), Faculdade de Ciências e Letras (FCL), Câmpus de Assis, Laboratório de Matemática Aplicada, Assis, SP Brazil; 2 Institut de Biologie de l’École Normale Supérieure de Paris, Paris, France; 3 In Vitro Brasil SA - Mogi Mirim, Mogi Mirim, SP Brazil; 4 Universidade Estadual Paulista (Unesp), FCL, Câmpus de Assis, Laboratório de Micromanipulação Embrionária, Assis, SP Brazil

## Abstract

Morphological analysis is the standard method of assessing embryo quality; however, its inherent subjectivity tends to generate discrepancies among evaluators. Using genetic algorithms and artificial neural networks (ANNs), we developed a new method for embryo analysis that is more robust and reliable than standard methods. Bovine blastocysts produced *in vitro* were classified as grade 1 (excellent or good), 2 (fair), or 3 (poor) by three experienced embryologists according to the International Embryo Technology Society (IETS) standard. The images (n = 482) were subjected to automatic feature extraction, and the results were used as input for a supervised learning process. One part of the dataset (15%) was used for a blind test posterior to the fitting, for which the system had an accuracy of 76.4%. Interestingly, when the same embryologists evaluated a sub-sample (10%) of the dataset, there was only 54.0% agreement with the standard (mode for grades). However, when using the ANN to assess this sub-sample, there was 87.5% agreement with the modal values obtained by the evaluators. The presented methodology is covered by National Institute of Industrial Property (INPI) and World Intellectual Property Organization (WIPO) patents and is currently undergoing a commercial evaluation of its feasibility.

## Introduction

According to the Agriculture Administration, the Brazilian herd of cattle is currently estimated to be approximately 215 million livestock units. Brazil produces the second largest worldwide herd and has been the leader in meat exportations since 2004 and in sales in more than 180 countries. This activity occurs in all Brazilian territory, and the gross value of production shows the important social and economic role of cattle for the country^1^. Brazil also leads the global *in vitro* production of bovine embryos, a segment of the industry that is of utmost importance for international and national improvement in cattle genetics and productivity.

In a typical commercial production chain, cattle embryos are produced *in vitro* and transferred to synchronized receptors when they reach the blastocyst stage^2^. The pregnancy rate (*i.e*., success rate) is directly dependent on the quality of the transferred embryos^3^. In the bovine species, there is an embryo classification system that is recommended by the International Embryo Technology Society (IETS), which is based on morphological evaluation and establishes three quality grades: excellent or good, “1”; fair, “2”; or poor, “3”^3^.

This embryo morphological analysis is performed by optical microscopy, and the grading of the embryo depends on the accuracy and experience of the embryologist to evaluate the variables that affect the development and pregnancy establishment potential of the embryo^3, 4^. Moreover, this morphological analysis does not measure any objective variables to determine the embryo classification; thus, it is subjective and has low reproducibility^5^. Indeed, the same embryo can be classified with different degrees of quality by different embryologists (inter-evaluator error) or even by the same embryologist (intra-evaluator error), especially in cases when the quality grade is borderline^6^.

Several methods have been or are being developed to provide a better solution for embryo classification, including a semi-automatized image segmentation process with the use of artificial intelligence (AI) for human embryos^7^, an automatic segmentation procedure of bovine embryos, but without the use of AI^8^, a semi-automatized grading method of human blastocyst using a support vector machine^9^, embryo metabolism analysis, cellular respiration measurements, the use of *zona pellucida* birefringence, microRNA profile determination, analysis based on logistic regression and evaluation by time-lapse video (reviewed by ref. 10). However, none of these methods are totally effective, and, despite being subjective and old, visual morphological analysis is still widely used^4, 6, 11^.

Recently, there have been attempts at creating a method based on digital image processing to determine the viability of human embryos by detecting blastomeres^12, 13^ or trophectoderm^14^. Additionally, using processing and digital image analysis in the quality evaluation of mouse blastocysts, a previous study used an artificial neural network technique with significant success^15^. However, as far as we can determine from the studied literature, a classification method using digital image processing has not been applied to bovine blastocysts.

In brief, an artificial neural network (ANN) is a system that solves problems by simulating the operation of a set of biological neurons. In particular, this artificial intelligence technique is suitable for solving nonlinear problems by using interconnected variables^16–18^. Its use is recommended in tasks in which traditional computational methods are limited; that is, when a large power of adaptation and generalization is required for data classification^19^. Therefore, the ANN is an intelligent system that can elucidate a complex problem through supervised learning. ANNs are already widely used in solving problems related to image processing^20, 21^. Thus, the technique is potentially suitable for blastocyst morphological classification from two-dimensional images, as previously described^15^.

However, there are some limitations in the classifiers that were developed based on ANNs; these barriers mainly include the development of the best network architecture (*i.e*., the number of layers and neurons as well as their weights)^22^.

Genetic algorithms (GAs) are computational methods based on natural mechanisms of evolution and genetics, and are used to solve a combinatorial problem^23^. Studies such as ref. 24 have demonstrated the successful use of GAs to determine the most efficient ANN architecture configuration. Furthermore, the use of GA has been effective in prediction models^25, 26^.

In this work, we use digital image processing to assess bovine blastocyst characteristics based on artificial intelligence techniques such as GA and ANN. We aim to classify embryo quality objectively and reproducibly using mathematical variables extracted from digital images. With this method, classification errors obtained with current subjective classification methods should decrease and the predictive value of gestational success could increase if conjugated with a good choice of the recipient of that embryo.

## Methods

### Embryo image capture and embryologists’ classifications

For the software development, a database containing 482 images captured at the company *In Vitro* Brasil (Mogi Mirim, SP, Brazil) of bovine blastocysts produced *in vitr*o (7 days post-insemination) was used. The images were obtained using an inverted microscope Olympus IX71 at 32x magnification and the software Lucam Capture v6.30; images were stored in JPG format in 8-bit colour (RGB) at a resolution of 1280 × 1024 pixels. Each image contained only one embryo, which was approximately centred in the visual field. To capture the image, the position of the blastocyst was standardized such that the plane of focus was in the largest diameter of the embryo and the inner cell mass (ICM) was perpendicular to the focal plane (*e.g*., Fig. S1 images *b*, *d* and *f*). We excluded any ICM image capture with a tangential or angled focal plane. An alternative capture was attempted in which the ICM was positioned parallel to and totally at the focal plane (Fig. S1 images *a*, *c* and *e*); however, in a pilot evaluation, this approach was shown to be disadvantageous in comparison to standard capture. Furthermore, a pilot experiment (data not shown) was conducted to evaluate the benefit of using two images captured from the same embryo in the ANN training (*e.g*., images *a* and *b* in Fig. S1). In this pilot experiment, the use of two images did not provide any benefit; therefore, this approach was standardized using a single image of the blastocyst and with the ICM perpendicular to the plane of focus.

Three experienced embryologists from the company (MFA, TGA and BLC), who were responsible for routine laboratory work, classified each image into three quality grades according to the IETS standards^3^. The blastocyst classification (n = 482) was distributed as follows: 113 images were classified as grade 1 (excellent/good); 175 were classified as grade 2 (fair); and 194 were classified as grade 3 (poor). After the blastocyst classification, the mode of the three evaluations was calculated as the main variable for use in the ANN training.

### Image processing

The digital image processing is an essential step to analyse and extract information from the blastocyst images. This information was used to obtain the input and output variables that were presented to the neural network during its training. The digital image processing comprised a series of techniques that have been widely used in solving several problems. This processing ranged from improving photographs to computer vision^27^.

### Image standardization

Each laboratory applies a different standard to capturing images; therefore, standardizing these methods has become indispensable. In addition to the resolution and formatting differences, different batches of images can have distinct lighting characteristics (Fig. S2).

For the segmentation and extraction of information to find similar conditions independently of the images’ original features, all the factors must be considered. All of the algorithms were developed using the MATLAB^®^ platform^28, 29^ and enabled the automated analysis of the images without the need for user intervention.

For standardization, the software consecutively followed the steps of image import, conversion to greyscale, resolution and proportion adjustment, and intensity adjustment (Supplementary Section 1).

### Blastocyst image segmentation

Once standardized, the images underwent a segmentation process, whereby the images were properly isolated from the background and were then subjected to the information extraction techniques. The steps used in the segmentation algorithm are described in detail in Supplementary Section 2.

### Texture analysis

The image texture was defined by repeated random regular patterns in a region of the image that provided information on the surface structure^30^. This variable is considered an important characteristic that is used to identify the regions of interest in an image^31^.

Among the statistical methods used to analyse the textures in images, the Grey Level Co-occurrence Matrix (GLCM) is considered to be among the most efficient^10, 30, 32^. GLCM describes the spatial distribution of the intensity values of the pixels by considering a determined distance and angle, which makes it possible to recognize and classify textures. Each matrix is the probability of two neighbouring pixels (one with intensity *i* and another with intensity *j*) at a determined distance *d* and angle *θ*, which forms *P* = (*i*, *j*, *d*, *θ*). For the calculation of GLCM, the image intensity was adjusted to have only 8 shades of grey. The image seen in Fig. S5 was used to generate the GLCM values listed in Table S1.

### Watershed Transform

The image segmentation step has a substantial influence on the independent interpretation of different regions. Two main approaches can be used for segmentation: detecting edges, which delimits a region, or searching for regions that have similar pixel intensities. The Watershed methodology searches the targeted image using the second strategy^33^. The Watershed transform proposes a morphological approach to the image segmentation problem through its interpretation of pixel intensities as surfaces, in which the grey levels of each pixel determine the height of a given region. Based on this concept, drainage basins, which are defined by regions of local minima and their domain regions, can be identified^34^.

Figure S9 shows an example of blastocyst segmentation by the Watershed transform. For visualization purposes, each region was assigned a random colour. The ICM has the largest area after segmentation, probably because it is a relatively homogeneous dark region. Therefore, the largest segmented area was used as an ICM mask in the variable extraction steps.

### Variable extraction

Once the embryo was properly standardized and isolated, an information extraction step was conducted (*i.e*., a numerical vector that represents the original image). Then, the variables obtained were used as inputs for the ANN technique.

All of the information extraction steps were performed automatically and without the need for user intervention. After the blastocyst image standardization and segmentation, the variables were extracted and used to determine the input vector for the ANN. To denominate the variables, the notation ER was used to refer to the blastocyst image version with an expanded radius by 5 pixels, RR was used to refer to the reduced radius by 40 pixels, and TE was used to refer to the difference between the two radii (Fig. S8). For a complete description of the chosen variables, see Supplementary Section 3. The 36 chosen variables were sufficient to extract all of the relevant information for the representation of the analysed bovine blastocyst image.

### Collinearity analysis

After the variable extraction was performed as described above, collinearity analysis was performed. This variable analysis was used as the input for the ANN, which is a common and recommended practice^22^. Using this analysis, it was possible to eliminate those variables that were correlated with one another (*i.e*., redundant), a factor that could lead to an unwanted predisposition in favour of certain variables over others.

It was also possible to determine the Variance Inflation Factor (VIF), which represents the degree of independent variable multi-collinearity when compared with the other independent variables. As a rule^35^, collinear variables can be considered to be those with higher VIF values than 10. The VIF value can be calculated per Equation 1, in which *Ri* is the determining coefficient of the regression of a variable *i*.

1 $$VIP\,\,=\,\,\frac{1}{1\,\,-\,\,{R}_{i}^{2}}$$

In this case, 13 iterations were performed until all of the variables had a VIF value of less than or equal to 10. Thus, at the end of the collinearity analysis, 24 variables remained for use in the ANN (Correlation RR, Energy RR, Correlation TE, Homogeneity TE, C1, Mean C1, C2, Mean C2, C3, Mean C3, C4, Mean C4, Sum, Mean ER, Mode RR, Mean Count RR, Bright RR, Mode TE, Mean Count TE, Bright TE, WSN, Convex ICM, Eccen ICM, and Mean ICM). Their mathematical and biological aspects are described in Table S2 (Supplementary Section 5).

After the variables that model the bovine blastocyst images were defined, the techniques of ANN and GA were applied.

### Artificial neural network

For the ANN learning process, the Backpropagation algorithm was used^16, 18^. The database (n = 482 blastocysts images) was divided into training, validation and test, with, respectively, 70, 15 and 15% of the images and following an already established validation standard^36–40^. Several transfer functions that perform the passage of information between neurons were used (logsig, purelin, tansig, hardlim, tribas, radbas and satlin^28^), training functions (trainrp, trainscg, traincgf, traingdx and traingdm^28^), hidden layers (between 1 and 3), and the number of neurons in each layer (between 10 and 120, ref. 17).

The accuracy of the obtained ANN was verified according to the error between the real values (the mode of the embryologists’ evaluation) and the values obtained by the ANN^16, 17, 41, 42^.

However, there is no standard method for obtaining the best architecture (the number of neurons in each layer, number of layers, training and transfer functions) of the ANN for a solution to a problem^43^. Thus, the GA technique was used to improve the efficiency of determining the best ANN architecture for our problem^23^.

### Genetic algorithm

In this study, we considered that the population to be studied by the GA was formed by different ANNs, and the goal was to obtain networks that had the lowest error in the blastocyst image classification. The GA technique developed in our study considered the creation of an ANN initial population with different architectures (individuals), which was randomly generated and composed of 100, 200 or 300 individuals.

Each one was defined by a “genetic code”, in other words, specification by 9 different genes (the number of neurons in the first, second and third hidden layer; the transfer function for the first, second and third hidden layer; the transfer function for the output layer; the training function to be used; and the quantity of hidden layers to be used). Each ANN was trained and tested, and their success percentage for the degree of classification was assessed. Therefore, the ANN architecture was defined in terms of its accuracy. A flowchart of the GA is shown in Fig. S10.

In the next step, a selection process according to elitism was used. In this process, individuals were organized based on the accuracy obtained by the ANN in the embryo classification of the test data. After the selection step, 10% of the fittest individuals (*i.e*., with lower misclassification) were used in the next generation.

In the replication and replacement step, beyond the 10% of the fittest individuals from the previous generation, 60% of the population consisted of new individuals added from the technical application of crossing-over and mutation among the individuals of the population (which yielded a maximum of 5% for the mutation effect). The remaining individuals (30%) were generated randomly while respecting the conditions that stemmed from the initial population. After selection, replication, and replacement, there was a new population from which the best ANNs were saved. This cycle was termed a generation.

On average, 1000 generations were produced for each population created. When we determined the number of individuals in the initial population (100, 200 or 300 individuals), this number of individuals remained unchanged until the end of the 1000 generations. The entire procedure was performed 500 different times for 100, 200 and 300 individuals.

### Further statistical analysis

Specific techniques were used to test whether the data obtained during the information extraction steps and database classification were correctly interpreted.

Receiver Operator Characteristic (ROC): One way to analyse the ANN results for pattern recognition is through a ROC curve. For ROC, two values are required: 1) the ratio of true test positives to total positives (TPR = True Positive Rate), or the Sensitivity; and 2) the ratio of false positives to the total negative (FPR = False Positive Rate), or the Specificity. The ROC curve is constructed using a diagram that represents the sensitivity values per the proportion of false positives given by (1 - specificity) for a diverse set of cut points^44^.

Confusion Matrix: This methodology is used to analyse the multiclass system results. Through this method, it is possible to observe the intersection between the data provided by the classifier (ANN system) and the original feedback rating (mode of the embryologists). Thus, we can obtain the overall accuracy of the system as well as the accuracy for each class (*i.e*., each quality grade)^45^.

Kappa index: The Kappa statistic, or coefficient, is often used for measuring the agreement between two or more evaluators when there is a degree of subjectivity in their interpretations. A Kappa index of 1 indicates a perfect agreement between the evaluators, and a value of 0 indicates that the evaluation by the observers is virtually random^46–48^. To evaluate the Kappa index, a sample of 10% of the image database (n = 48) was used twice in separate evaluations, and three embryologists reclassified the previously classified images in a blind test. Thereby, we measured the intra-evaluator disagreement rate, which, together with the rate of disagreement among evaluators (classification of the total images database, n = 482), provided the profile of the embryologists’ subjectivity.

## Results

From the analysis of 482 blastocyst images, the three best ANN architecture parameters obtained using the GA technique are presented in Table 1. The ANN was successful for grades 1, 2 and 3 of the embryo quality (Table 1) when referring to the test data (15% of the total sample), and they were not used for training the ANN (*i.e*., the test data were used in a blind test).

**Table 1:** Parameters of the best ANN architectures obtained using the GA.

Parameters		ANN Architecture		
		1	2	3
Neurons	1st layer	80	95	59
Transfer function	between input and 1st layer	tribas	radbas	Tribas
	output layer	purelin	purelin	Purelin
Training function		trainscg	traincgf	Trainscg
Success grade 1 (%)		71.4	66.7	65.0
Success grade 2 (%)		71.4	78.3	70.8
Success grade 3 (%)		81.1	80.6	89.3
Total success (%)		76.4	76.4	76.4
mse (mean square error)		0.116	0.082	0.126

**Figure 1:**
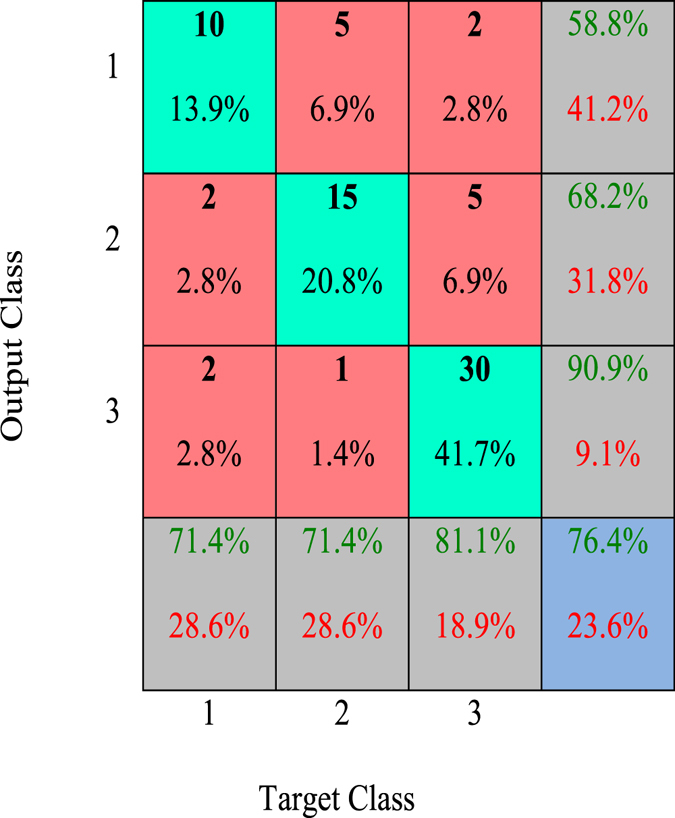
Confusion matrix for the ANN test data with architecture 1.

Considering only the test data, the confusion matrix for architecture 1 (Table 1) is shown in Fig. 1. In this image, the green and red cells represent the successes and errors in the ANN classification, respectively, compared to the mode of the embryologists’ evaluation.

In the confusion matrix, it can be observed that the ANN correctly classified 76.4% of the embryos in the test data (blue cell, Fig. 1). There were only four images that had serious classification errors (5.6% of 72 images); these errors occurred when the embryologist classified the embryo as grade 1 and the ANN classified the embryo as grade 3 (and vice versa). Nevertheless, the technique demonstrated robustness in the consistency of the classification.

**Figure 2:**
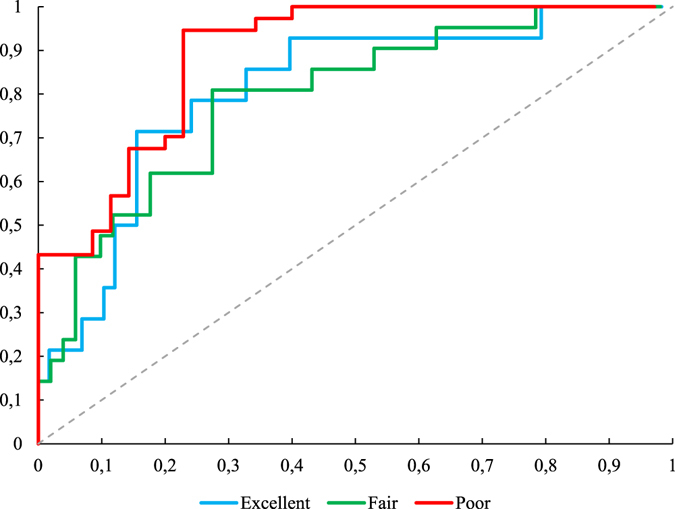
ROC curve for the ANN test data with architecture 1.

The ROC curve obtained for the test data set (Fig. 2) showed excellent performance for the ANN; that is, the curve is well placed on the northwest region of the graph.

**Figure 3:**
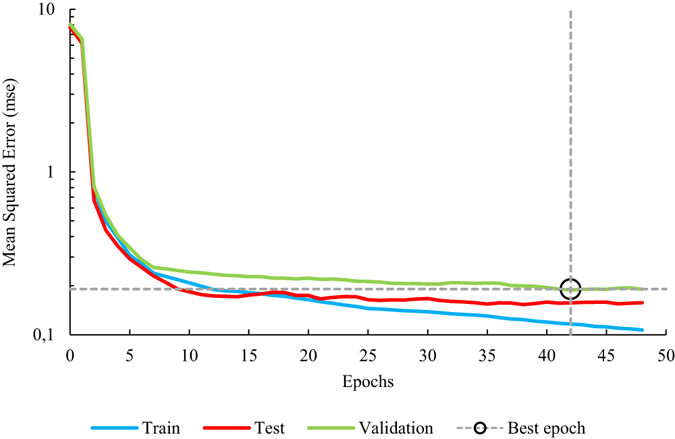
Dynamic performance of the ANN with architecture 1.

The performance of the ANN architecture 1 on the totality of the generations is shown in Fig. 3.

**Figure 4:**
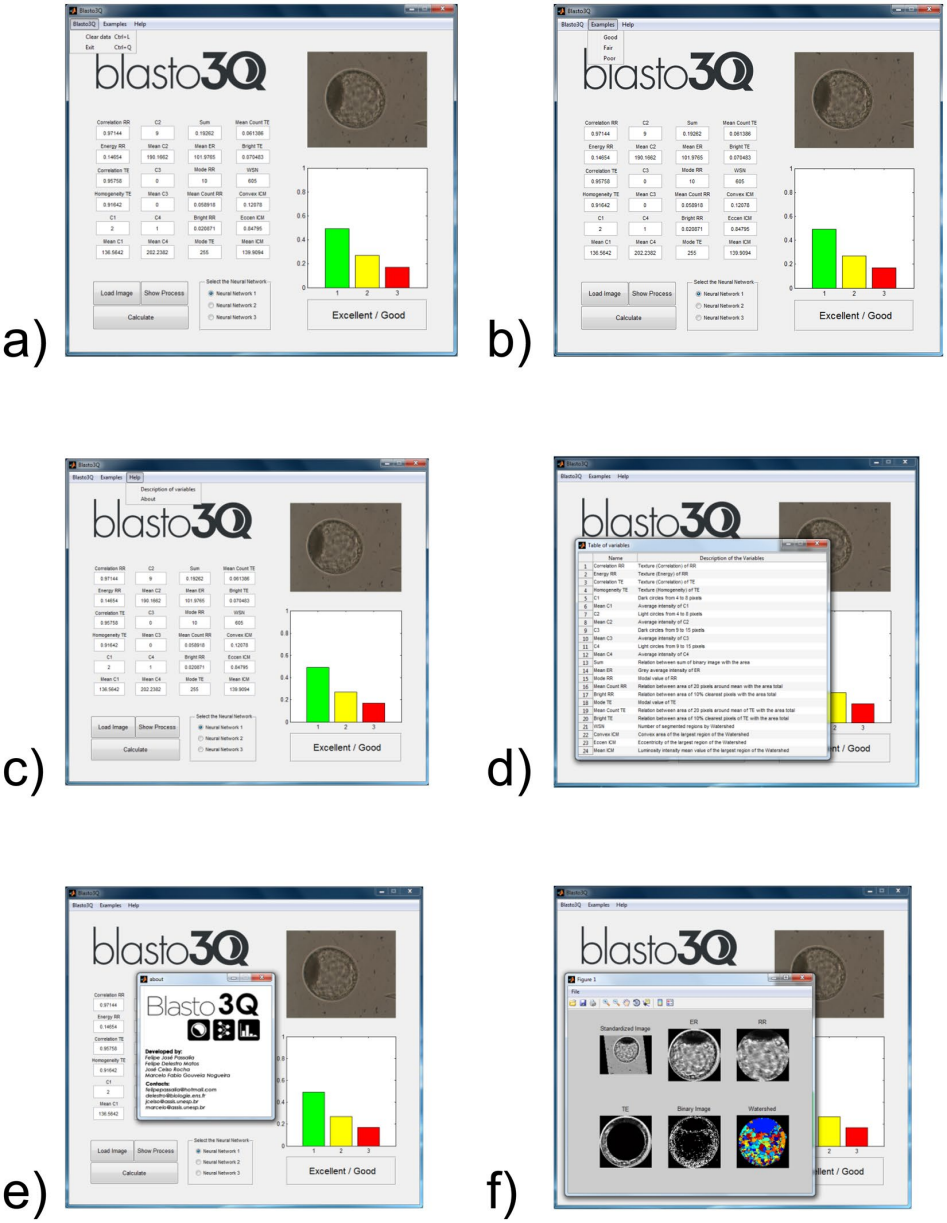
Tabs and buttons of the user-friendly interface. (**a**) Tab with options to clear the data or to close the interface; (**b**) tab with three images as standard models of excellent/good, fair, and poor; (**c**) tab with the variables and about the authors; (**d**) tab with the nominal description of the 24 variables; (**e**) tab with the authors’ details and e-mail correspondences; and (**f**) Standard, ER, RR, TE, Intersection and Watershed images of the selected embryo.

This work resulted in two different interfaces, which were an interface developed using the MATLAB^®^ platform and an interface developed using a multiplatform approach for online purposes. For both interfaces, we called the program “Blasto3Q”, which refers to the three qualities of the blastocyst evaluation. In the first case, a graphical user-friendly interface was created. In this way and in accordance with the recommendations for the commercial use of the program, the potential user can interact with the program in a fast and intuitive way. This interface (Fig. 4) contains a description of the 24 variables that are extracted from the image and analysed by the ANNs; moreover, it classifies the same loaded image through the best three obtained ANNs (Architectures 1, 2 and 3, Table 1).

In addition, there are three superior tabs that are available: in the first tab (Blasto3Q), there are options to clear the data or to close the interface; in the second tab (Examples), there are three images for standard models of grade 1 (excellent/good), 2 (fair) and 3 (poor); in the third tab (Help), one sub tab provides a nominal description of the variables that are used, and the other sub tab provides the authors’ names and corresponding e-mail addresses. Moreover, in the inferior region of the interface, there is a button (Load Image) with which the user can select an image from a personal file for the software analysis, and a button (Show Process) that makes available graphical representations of the Standard, ER, RR, TE, Intersection and Watershed variables of the selected image. Finally, by clicking the Calculate button, the software classifies the image of the selected blastocyst and the ANN provides a histogram, which appears at the bottom right of the open window. In Fig. 4, all of the tabs and buttons are shown. The performance of the three ANNs of the interface is shown in Fig. S11.

The second developed interface allows the on-line use of Blasto3Q via a cell phone (smartphone) or a PC. In these cases, a server PC receives the embryo image via upload and provides the results to the user (see Fig. S12 and the link to Blasto3Q in Supplementary Section 6).

## Discussion

From the general results obtained, the applied artificial intelligence technique which combined the ANN and GA methods, was successful (76.4% accuracy) in classifying the *in vitro* bovine blastocyst quality. The application of the GA aimed to select the ANN that had the best performance in bovine blastocyst classification using 1000 generations. To emulate the Darwinian principle of natural selection via crossing-over, mutation and migration, there was an evolutionary convergence of all the selected ANNs (Table 1) because they had only one hidden layer, a transfer function for the first layer (tribas or radbas), only the purelin function for the output layer, and the training functions trainscg or traincgf.

In this work, the ANN training was considered to be the mode among the evaluation of three experienced embryologists of *In Vitro* Brasil. Thus, the output value from the ANN for a blastocyst image was compared to the mode from the embryologists’ evaluation of this same image. The option for the mode of the three evaluations (as a comparison standard) avoids the bias of using a single evaluation as the standard for the ANN training^15, 49^. Although the embryologists were experienced, the Kappa index was 0.571 (482 images, P < 0.001), which shows that there was a moderate or weak agreement among their evaluations; similar discrepancies have reported by others^4, 11^. In ref. 6, a Kappa index of 0.42 was reported (where 1.0 indicates complete agreement among the evaluators) for the image evaluations of bovine embryos produced *in vitro*. Thus, the result obtained using artificial intelligence methods (Table 1), in which the best ANNs had 76.4% accuracy, was even more satisfactory because the Kappa index of the three ANNs was 0.616 (482 images, P < 0.001) and there was a low percentage of serious errors (5.6%). This type of error (differing by ≥2 degrees of quality) has been reported to be 10%^49^ when two of five experienced embryologists evaluated images of bovine embryos.

In the interface provided to the user, the best three ANNs are made available. Although all of the ANNs had similar accuracy, they varied in the classification of the embryos in a manner that mimicked the variations among experienced embryologists. For example, there were subtle differences in the best result of a certain grade. Thus, the best ANNs to classify the quality grades 1, 2 and 3 were architectures 1, 2 and 3, respectively (Table 1). In this way, the interface mimicked the increase in the robustness of the classification when more than one embryologist assesses an embryo. When evaluating the same image with the three ANNs, the user can discover whether there is consensus among the ANNs or, in the absence of full agreement, opt for the classification that is provided from two of the three networks.

As a blind test to determine the intra-evaluator variation (*i.e*., the subjectivity of the evaluation by an embryologist), 48 images were chosen (10% of the total sample and the more complex images, which were evaluated as borderline between two grades). In most of these images, the embryologists did not reach complete agreement regarding the blastocyst classification. After two new blind classifications of the 10% sample (three evaluations in total, considering the first sample with 482 images), a low intra-evaluator agreement was observed (35, 48 and 52%) as well as Kappa indexes of 0.28, 0.41 and 0.47, respectively (P < 0.001). A similar event was reported by a previous study^11^, in which the investigators observed a non-optimal intra-embryologist Kappa index. When such a reclassification (a sample of 48 images) was compared to the original mode value used for the ANN training, there was a success rate of 54, 52 and 50% for the embryologists. However, the best obtained ANN reclassified the 48 images with an accuracy of 87.5% compared to the original mode value. Additionally, when the intra-ANN agreement was evaluated, there was 100% agreement and a Kappa index of 1.0 when comparing the grade scored for the same image in each of the three analyses. Thus, these results support the robustness and low subjectivity in the grading of blastocyst images by the ANNs.

Both of the graphical interfaces that were created allow for friendly use of the program and differ from other attempts to semi- or fully automatize embryo evaluation^7–9^ in the absence of such an interface (Fig. 4).

In both interfaces, the result is shown as a histogram, which presents the relative magnitudes in each of the quality grades (excellent/good, fair, poor). Thus, the user can interact with the tool to check whether their personal evaluation of the same image is consistent with the results presented by the chosen ANN. Moreover, the subtle information provided by the histogram is the weight of the highest and second grades; that is, the reliability of the highest grade provided by the network or a decision on an intermediate grade when the weights for two grades are very close. Similarly, a user-friendly interface is available only on devices that have time-lapse embryo monitoring (Primo Vision^TM^, Eeva^TM^ Test and Embryoscope^®^) and built-in software for the evaluation of the kinetics and cleavage symmetry^50^.

As far as we can determine, this study is the first to develop a fully automated ANN-based software for the evaluation of mammalian embryos. Although ref. 7 used artificial intelligence (Levenberg-Marquardt neural networks) for oocyte and embryo classification, their method was semi-automated. In ref. 8, a non-supervised algorithm for the automatic segmentation of bovine embryos was proposed, but this algorithm did not use artificial intelligence or classify the embryo quality according to the criteria of the IETS. In ref. 9,73 human blastocysts were evaluated by a Support Vector Machine classifier, and the authors developed a semi-automated classification system.

Finally, the developed program can be used as a mobile application, in which a provider performs the analysis themselves and only sends the results to the phone; this application is in accordance with the current trend toward software mobility. In ref. 51, the authors developed an application associated with a single-ball lens to increase the compatibility of the image for use on mobile phones. This device enabled the evaluation of human semen samples, and the results for the sensitivity and specificity when compared to the standard method (CASA) were 87.5% and 90.9%, respectively.

The artificial intelligence technique that combines GA and ANN proved to be a powerful tool for the classification of bovine blastocysts. After the training with the mode of the embryologists’ evaluation, the program had a high accuracy and less subjectivity than the embryologists themselves; that is, high reproducibility when they classified the same embryo image three separate times.

The robustness of the combined technique with the ease of using an interface created for an interaction between the embryologist and the software can become a tutorial technological tool to be used both for academic purposes and in commercial laboratories. In these areas, which work on a large scale with the *in vitro* production of bovine embryos, the program could add a quality label and better tracking for the embryo. Currently, the program is protected by national (BR 10 2012 0319535 A2, ref. 52) and international (WO/2014/089647, ref. 53) patents. We did not overlook the potential for the adaptation of the current version of the program (to the bovine species) to be applied to the classification of human embryos. This potential could open prospects for its incorporation (built-in) into the existing commercial equipment used in embryo time-lapse monitoring.

## Electronic supplementary material

Supplementary information
