# Supplementary material for: A Method Based on Artificial Intelligence To Fully Automatize The Evaluation of Bovine Blastocyst Images
Source: Sci Rep. 2017 Aug 9;7:7659. doi: 10.1038/s41598-017-08104-9 (PMC5550425; doi:10.1038/s41598-017-08104-9)
Supplement: Supplementary file 1 — Supplementary information [file 41598_2017_8104_MOESM1_ESM.pdf]

SUPPLEMENTARY INFORMATION

A METHOD BASED ON ARTIFICIAL INTELLIGENCE TO FULLY AUTOMATIZE THE EVALUATION OF BOVINE BLASTOCYST IMAGES

José Celso Rocha[1], Felipe José Passalia[1], Felipe Delestro Matos[2], Maria Beatriz Takahashi[1], Diego de Souza Ciniciato[1], Marc Peter Maserati Jr[3], Mayra Fernanda Alves[3], Tamie Guibu de Almeida[3], Bruna Lopes Cardoso[3], Andrea Cristina Basso[3], Marcelo Fábio Gouveia Nogueira[4]

[1]Universidade Estadual Paulista (Unesp), Faculdade de Ciências e Letras (FCL), Câmpus de Assis, Laboratório de Matemática Aplicada, Brazil

[2]Institut de Biologie de l'École Normale Supérieure de Paris, Paris, France

[3]In Vitro Brasil SA - Mogi Mirim, Brazil

[4]Universidade Estadual Paulista (Unesp), FCL, Câmpus de Assis, Laboratório de Micromanipulação Embrionária, Brazil

Contents

1. Image standardization…………………………………………………….. 3
2. Image segmentation……………………………………………………….. 3
3. Complete description of the chosen variables…………………………….. 5
4. Supplementary Figures……………………………………………………. 10
5. Supplementary Tables…………………………………………………… 17
6. Link to the Blasto3Q online access………….……………………………. 20
7. References……………………………………………………………… 20

1. Image standardization

For standardization, the software followed the steps below:

1. Image importing: The image was imported into the MATLAB® platform, regardless of image format (BMP or JPG);

2. Conversion to greyscale: Colour information was not used in the interpretation of images and thus all images were converted to 8-bit greyscale. This process also rendered all subsequent steps faster since a decrease of the spectral image size occurs (from the three RGB bands to only one);

3. Resolution and proportion adjustment: In addition to different resolutions, the images also had different proportions. An image of 640x480 pixels has a proportion of 0.75 while for an image of 1280x1024 pixels the proportion is 0.8. Therefore, to only resize the images for one of the resolutions would distort the proportions of the other. We choose 640x480 pixels as default resolution, because it is the lowest standard and provides sufficient information for interpretation. Using the lowest resolution also increases the efficiency of the subsequent steps of the algorithm; besides it does not create minimum resolution limitations for the final software. Therefore, the 1280x1024 resolution was adjusted for ratio of 0.75 (by cutting an upper region of 64 pixels), and then resizing to 640x480 pixels. The proportions can be visualized in Figure S3;

4. Intensity adjustment: To minimize the effects of different illuminations, the images were submitted to a histogram adjustment, in which 1% of all information becomes saturated between light and dark pixels. This process also increased the image contrast, which facilitates the subsequent step of segmentation (Fig. S4).

2. Image segmentation

The following steps were used in the segmentation algorithm.

Image gradient: Initially we calculated the magnitude of the image gradient, and the edges were highlighted for subsequent steps. This operation detected edges in all directions, essential

characteristic for the circular shape of the embryo. The greater the intensity of the pixels variation, the greater the resulting magnitude value in the final gradient;

Binary image: In this step we calculated the binary image and selected the value of 128, as the intensity threshold (because it was an 8-bit image with 255 as the maximum intensity value for each pixel; Fig. S6);

Circular Hough Transform: The Hough Transform was originally used to detect straight lines, but adjustments allowed it to be used for the detection of any definite shape. In this work we used the Circle Hough Transform (CHT), which became widespread in circles detection processes in digital imaging[1]. Once the binary image was obtained, the Hough transform detects the embryo circumference by mapping the image and thus provides the isolated embryo background image. The algorithm performed the detection of circles in two stages: in the first stage, it searches by circles of radius between 100 and 150 pixels, then, it searches radius between 150 and 250 pixels for greater accuracy. These values were obtained after those steps by algorithm verification using the entire database. Therefore, both initial blastocysts (smaller) and expanded blastocysts (larger) can be correctly detected. At the end of both searches, in each image, the detected circles metrics are compared and the largest radius is used after the best circle is detected (Fig. S7);

Blastocyst isolation: After circumference detection, representing the blastocyst, we used this next step to isolate it. Three versions of the blastocyst image were generated. In the first, the radius of the circumference is increased by 5 pixels to make sure the *zona pellucida* is included (called ER); in the second, the radius was reduced by 40 pixels, in order to discard the trophectoderm, selecting the inner cell mass (ICM) and blastocoel for analysis only (called RR); and in the third version we obtained the difference between ER and RR. Thereby, only the trophectoderm region was isolated in the image (called TE; Fig. S8). The pixel values, which determined the expansion (ER) and the contraction (RR) of the blastocysts images, were obtained by assessment of the image database.

3. Complete description of the chosen variables

After image standardization and segmentation, the 36 variables described below were extracted. They determined the input vector for the ANN. The notation ER was used to refer to the blastocyst image version with expanded radius by 5 pixels, RR to refer the reduced radius by 40 pixels and TE to refer the difference between the two radii.

1) Contrast RR

GLCM determined variable. Contrast is the measurement of the intensity difference between a pixel and its neighbours across the entire image with a constant image of zero contrast. This is calculated by Equation $S1$

$$\sum_{i,j} |i-j|^2 p(i,j) \qquad (S1)$$

Where i, j and p are defined in the texture analysis item.

2) Correlation RR

Demonstrate the correlation between the image pixel in determined neighbour across the entire image. Values -1 or 1 shows a perfect correlated image, negative or positive respectively. Determined by Equation $S2$:

$$\sum_{i,j} \frac{(i-\mu_i)(j-\mu_j)p(i,j)}{\sigma_i \sigma_j} \qquad (S2)$$

3) Energy RR

Square of sum of the GLCM elements. An energy value equals to 1 correspond to a constant image. Represented by Equation $S3$:

$$\sum_{i,j} p(i,j)^2 \qquad (S3)$$

4) Homogeneity RR

Proximity measurement of the distribution of GLCM elements with the GLCM diagonal. The homogeneity value is 1 for a diagonal GLCM. Represented by Equation *S*4:

$$\sum_{i,j} \frac{p(i,j)}{1+|i+j|} \qquad (S4)$$

5) Contrast TE, 6) Correlation TE, 7) Energy TE and 8) Homogeneity TE

These variables follow the same principle of the variables 1, 2, 3 and 4, respectively. However, this is used as basis for the TE region instead of RR for the GLCM calculations.

9) C1

Hough transform for the dark circle detection with radius between 4 and 8 pixels. The final result is the quantity of found circles, with 0.935 of sensibility index (standard value used in the accumulative matrix of the transform).

10) Mean C1

Mean of luminous intensity of all circles detected in C1. The circle centres and the mean radius of them are used in the construction of a binary mask, which applied in the original image of the segmented blastocyst allows for the isolation of circles only. Then, the mean of luminous intensity in the isolated regions is calculated.

11) C2 and 12) Mean C2

These variables follow the same standard of the variables C1 and Mean C1, respectively. However, in the variables C2 and Mean C2 light circles are detected.

13) C3 and 14) Mean C3

These variables follow the same standard established of the variables C1 and Mean C1, respectively. However, circles of radius 9 to 15 pixels with 0.94 of sensibility are detected.

15) C4 and 16) Mean C4

These variables follow the same standard of the variables C3 and Mean C3, respectively. However, in the variables C4 and Mean C4 light circles are detected.

17) Radius

Calculated as half of the width of image ER.

18) Sum

A binary ER image is calculated, using the Otsu algorithm ref. 2 for the threshold detection. Next, the sum of all values from the binary image is calculated. Finally, this value is divided by the total area of the blastocyst.

19) Mean ER

Grey mean intensity of the pixels in ER.

20) Deviation RR

Standard deviation of the grey intensity of the pixels in RR, which is calculated by the Equation $S5$, where x is the image intensity vector and n the number of vector elements.

$$Std\ Deviation = \left(\frac{1}{n-1} \sum_{i=1}^{n} (x_i - \bar{x})^2 \right)^{\frac{1}{2}} \qquad (S5)$$

Where: $\bar{x} = \frac{1}{n} \sum_{i=1}^{n} x_i$

21) Mean RR

Calculated by the same way of Mean ER, but using the region of RR.

22) Mode RR

Mode value of RR, *i.e.*, the total value of luminous intensity more frequently in RR.

23) Dark RR

Initially, pixels with luminous intensity less or equal to 25, which is 10% of the limit allowed (remembering as it uses 8-bit values, the luminous intensity varies between 0 and 255). Then, this value is divided by the total area of the embryo.

24) Mean Count RR

All the pixels with luminous intensity between 10 pixels below and 10 pixels above than the mean intensity were counted. Then, this value is divided by the embryo total area.

25) Bright RR

Same as Dark RR, but using the pixels counting of intensity higher or equal to 230 (10% lighter of the image).

26) Deviation TE, 27) Mean TE, 28) Mode TE, 29) Dark TE, 30) Mean Count TE, and 31) Bright TE

These variables follow the same standard of variables 20 to 25, respectively. However, the TE image is used as basis to calculation.

32) WSN

Total number of regions found by the Watershed transform.

33) Area ICM

Area of the largest region found by the Watershed transform, corresponding approximately to the ICM area.

34) Convex ICM

Area of the smallest convex polygon, which has the largest detected region by the Watershed transform. Thereby, a reliable representation of the ICM real area is intended.

35) Eccen ICM

Eccentricity of the largest region detected by the Watershed transform. The value of zero would indicate a perfect circular area and the value 1 a line segment. Considering an ellipse that has the approximate shape of the largest region detected by the Watershed transform, Eccen ICM is calculated as being the relationship between the ellipse focus distance and the length of its largest axis.

36) Mean ICM

Luminous intensity mean value of the largest region detected by the Watershed transform.

4. Supplementary Figures

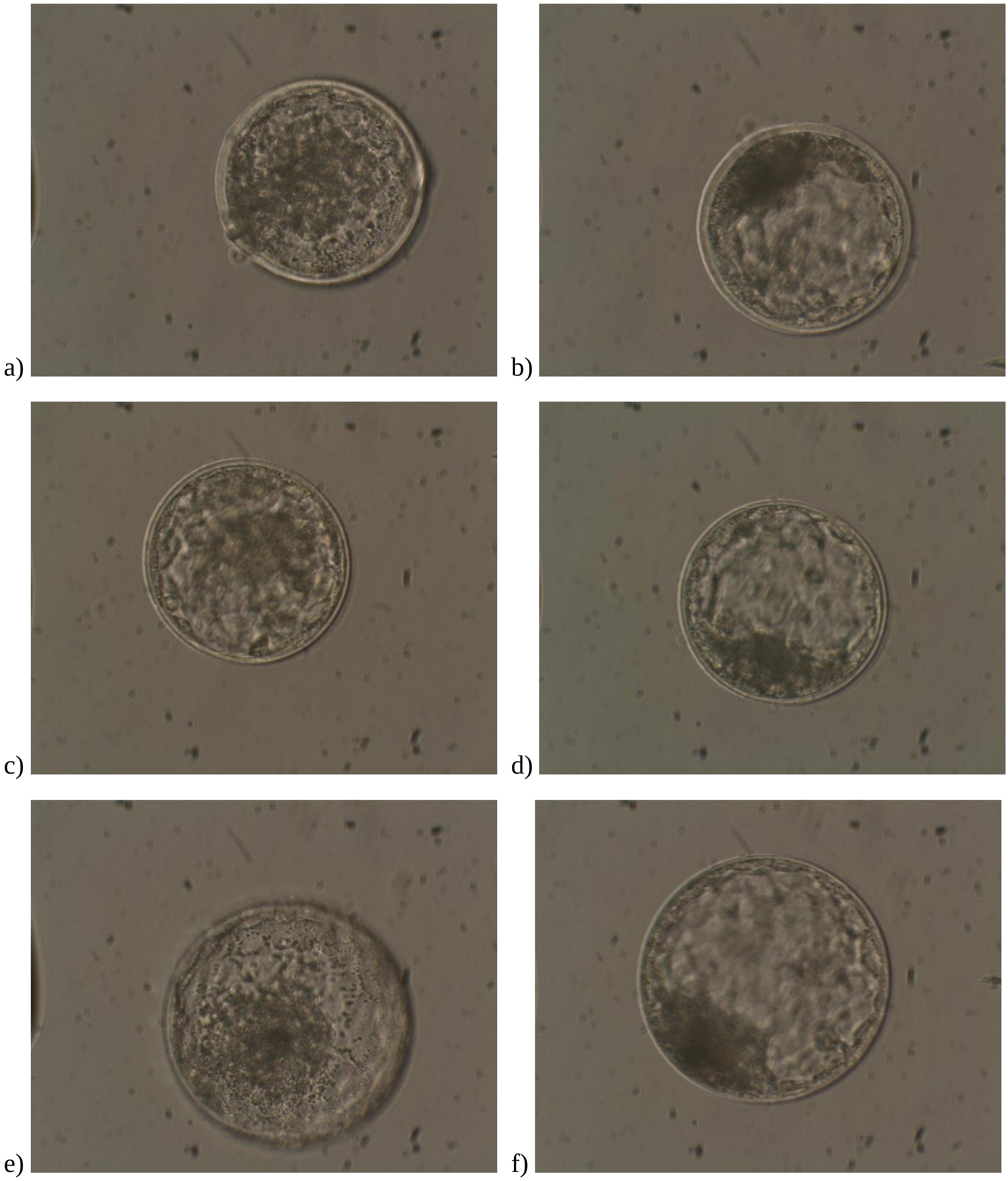

Figure S1. Images of embryos where the inner cell mass is positioned perpendicular to (b, d and f) or parallel and into (a, c and e) the focal plane. Three embryos are shown on each column and the same embryo is shown on each row.

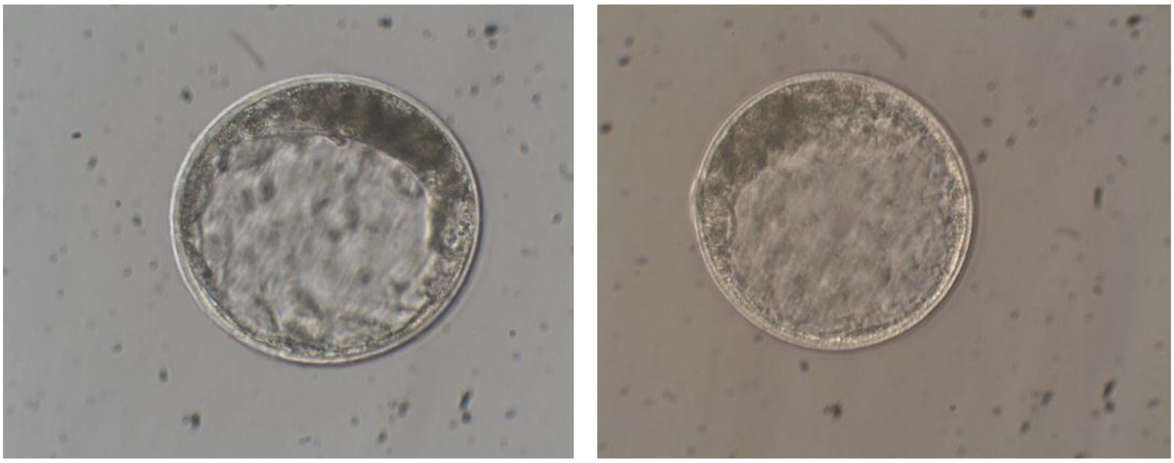

Figure S2. Image of blastocysts captured in different moments. Note the differences in light between the two images, which requires standardization by image processing.

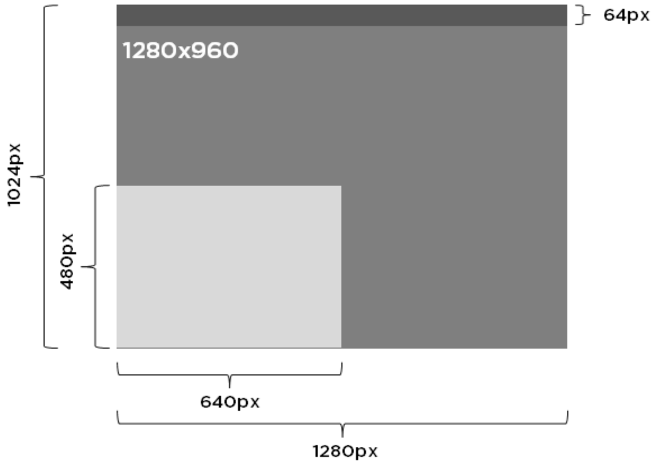

Figure S3. Scales and standard demonstration of the resolution and the proportion adjustment algorithm.

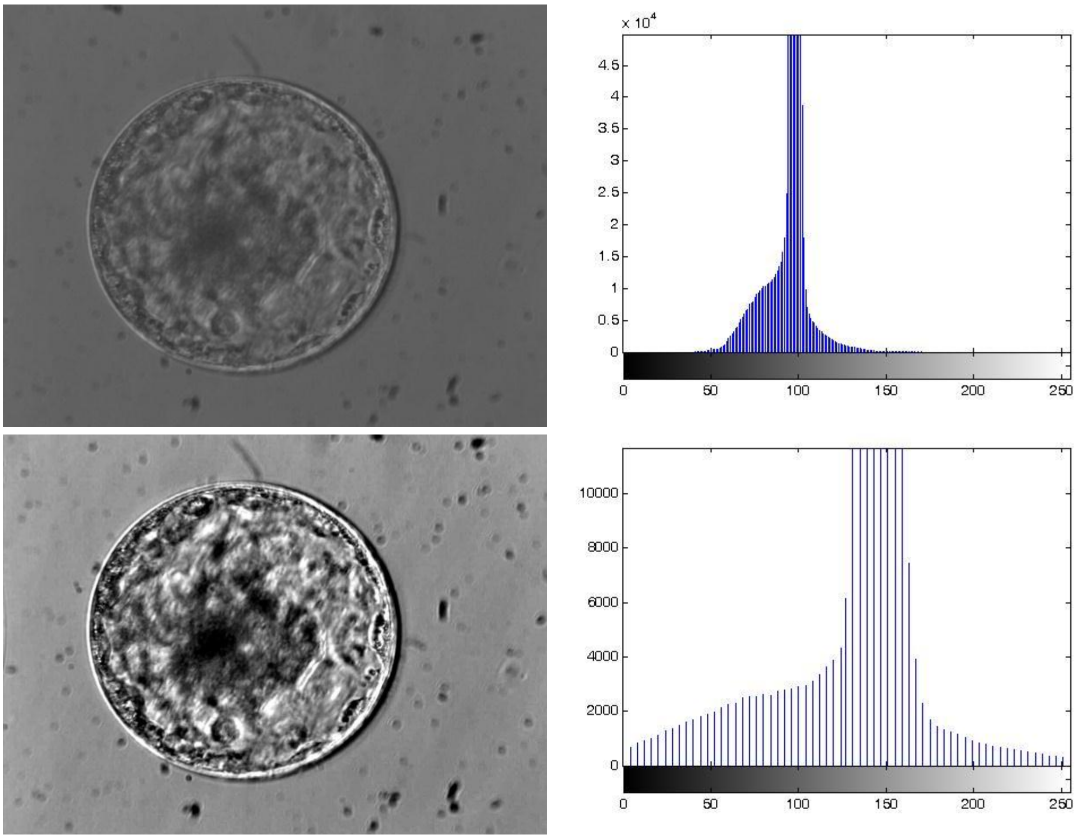

Figure S4. Images of a bovine embryo before (above) and after (below) the process of standardization. It is possible to visualize the correspondent histogram in the right of each image.

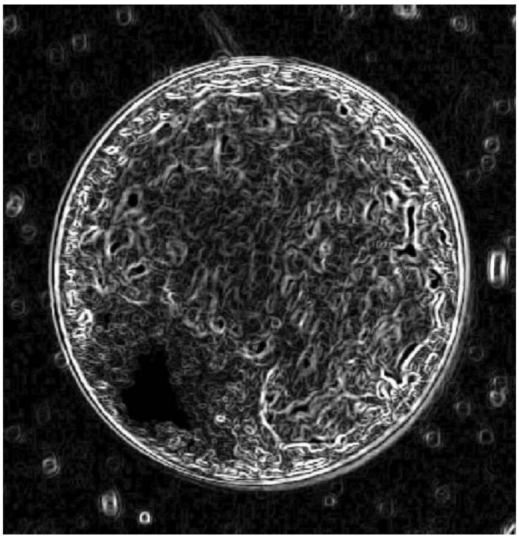

Figure S5. Resultant image from the GLCM calculation in a bovine blastocyst image after standardization.

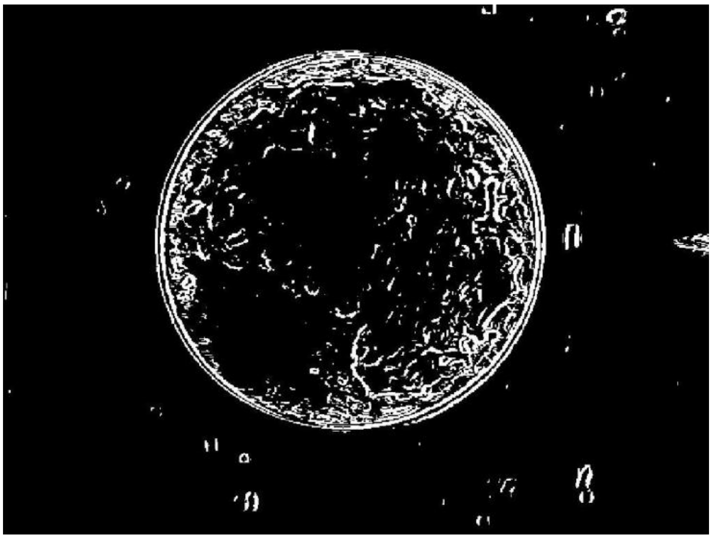

Figure S6. Binary image after standardization and gradient calculation. White pixels have value 1 and black pixels have value 0.

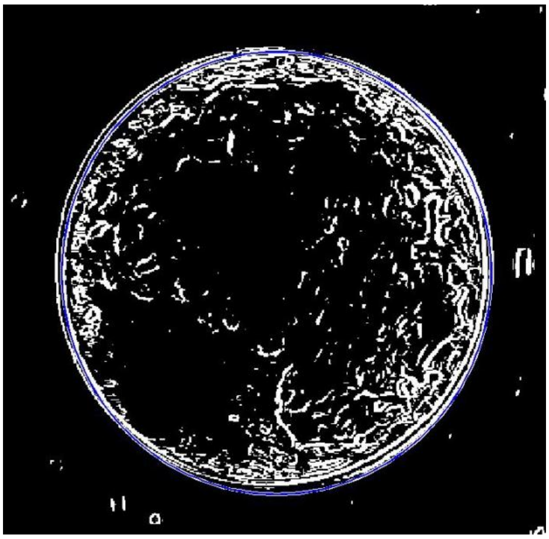

Figure S7. The best circle found by the Hough transform, represented by the blue line.

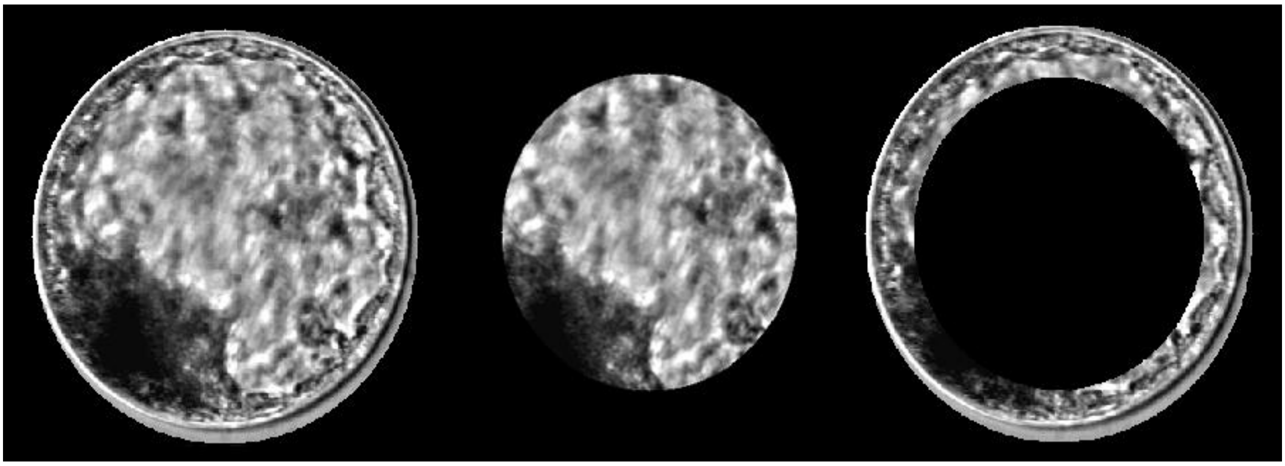

Figure S8. Final version of an isolated image of a bovine blastocyst. In the left, enlarged radius version in 5 pixels (ER); in the centre, reduced radius in 40 pixels (RR); and in the right, the differences between the two radii (TE).

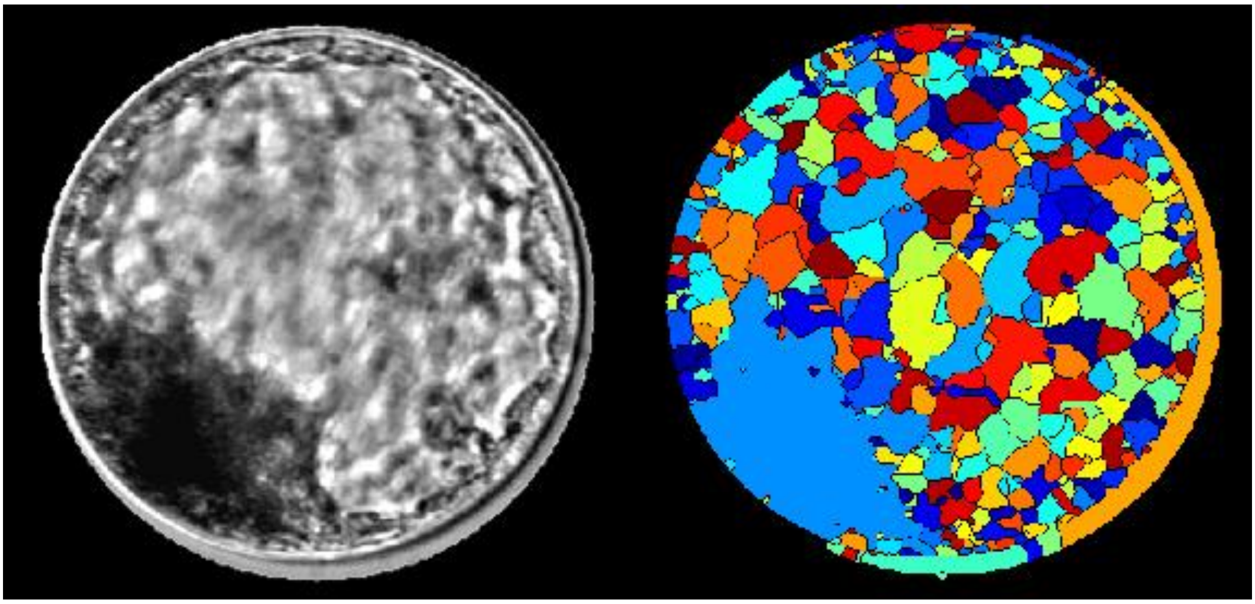

Figure S9. Example of a blastocyst image (left) and the segmentation by Watershed transform (right).

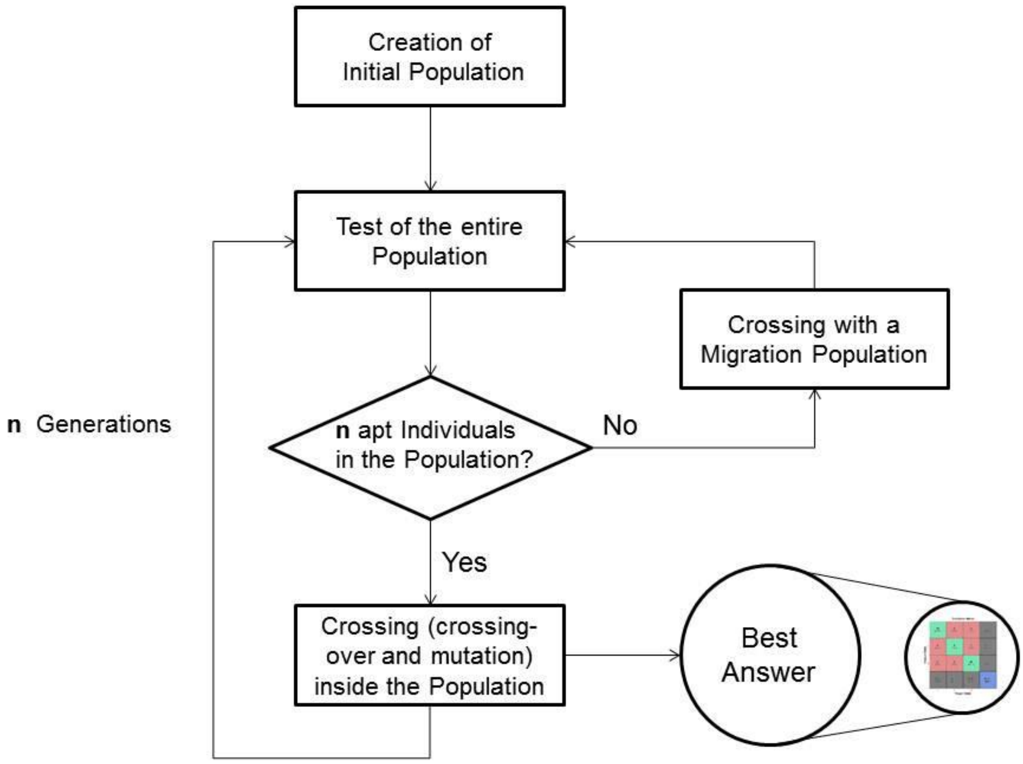

Figure S10**.** Performance flowchart of the genetic algorithm.

a) 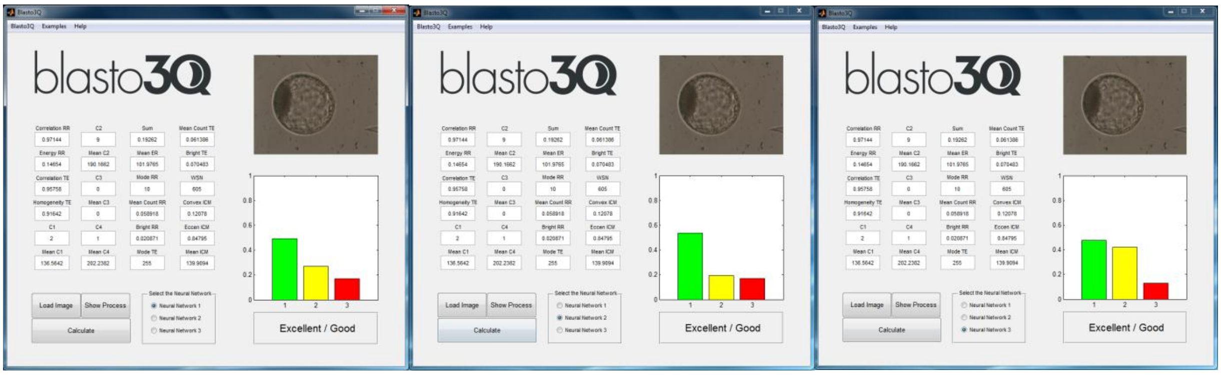

b) 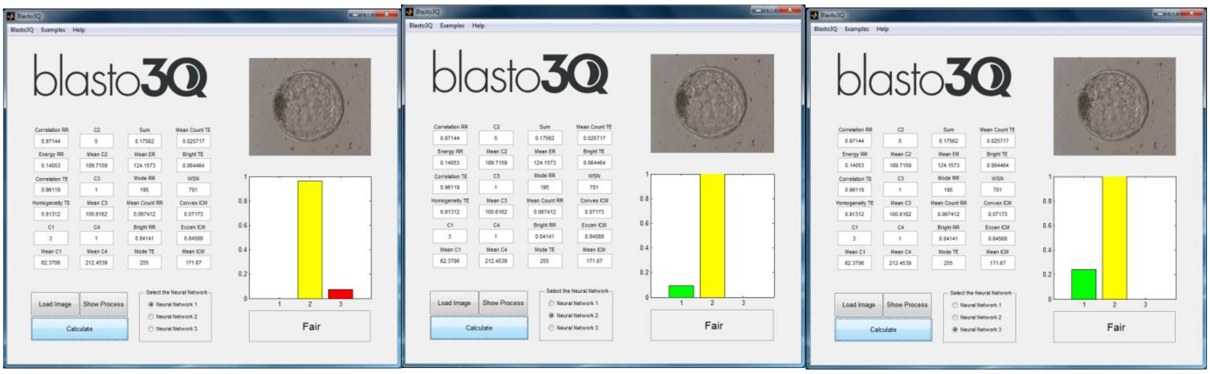

c) 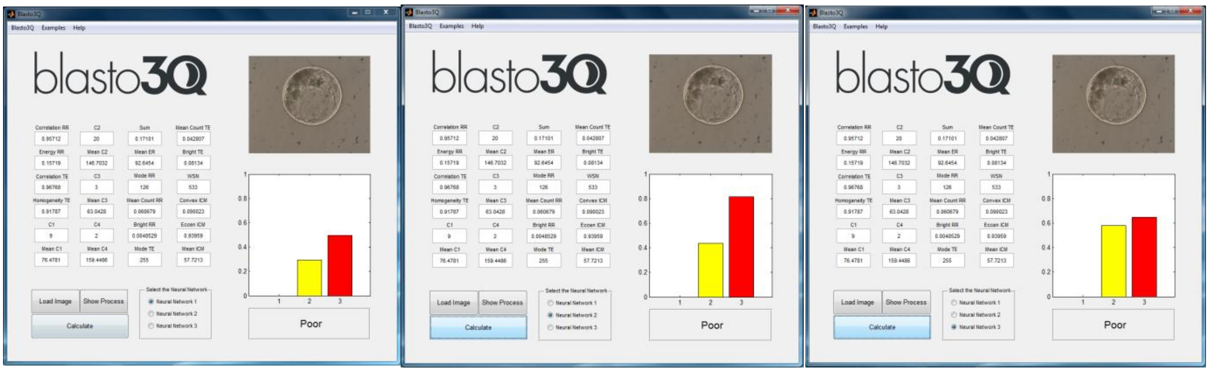

Figure S11. Interface of the three evaluations provided by different ANNs. a) interface analysing an embryo classified as excellent/good by the three ANNs; b) interface analysing an embryo classified as fair by the three ANNs; c) interface analysing an embryo classified as poor by the three ANNs.

5. Supplementary Tables

Table S1. Gray Level Co-occurrence Matrix from the figure S5.

	**1**	**2**	**3**	**4**	**5**	**6**	**7**	**8**
**1**	37449	1032	35	0	17	357	0	0
**2**	1003	5966	1541	74	2	0	0	0
**3**	64	1438	6766	2291	106	1	0	0
**4**	0	136	2080	10348	3076	96	3	0
**5**	84	14	231	2833	17050	3517	128	3
**6**	290	0	13	188	3375	19637	2233	167
**7**	0	0	0	5	226	2098	6114	1277
**8**	0	0	0	0	9	196	1242	5439

Table S2. Biological and mathematical description of the 24 quantitative variables used to the predictive evaluation by the software Blasto3Q.

Variable	Image processing step	Description
Energy RR and Homogeneity TE	GLCM	Statistical method used to analyse the texture of the image and considered to be one of the most efficient. Biologically they represent cellular homogeneity within the embryo image.
Correlation RR and Correlation TE	Mathematical operations	Mathematical operations. Also, they represent the homogeneity of the embryonic cells in the image.
C1, Mean C1, C2, Mean C2, C3, Mean C3, C4 and Mean C4	Hough Transform	It defines the circles around intact embryos in digital imaging. The variables identify rounded structures such as extruded non-degenerated blastomeres.
Sum	Otsu algorithm	Used in binary image calculation. It highlights the biological aspect of cellular edges with the more intense contrast.
Mean ER, Mode RR and Mode TE	Grey intensity	Pixel grey intensity of three versions of the blastocyst image: expanded radius by 5 pixels (ER); reduced radius by 40 pixels (RR); and the difference between the two radii (TE). They represent, respectively, the average grey intensity of the entire blastocyst (ER), the embryo without the

		trophectoderm (RR) and primarily the trophectoderm (TE).
Mean Count RR, Bright RR, Mean Count TE and Bright TE	Luminosity intensity	Variables defining pixels with various luminous intensity. They do not correlate with any biological aspect of the embryo.
WSN, Convex ICM, Eccen ICM and Mean ICM	Watershed Transform	Morphological approach to overcome the segmentation issue. It understands the pixel intensities as surfaces where light pixels are high and dark pixels are low. It uses variables that are related to the visual differences and similarities of each region from the embryo.

6. Link to the Blasto3Q online access

The link to the Blasto3Q is: http://186.217.161.71/blasto/

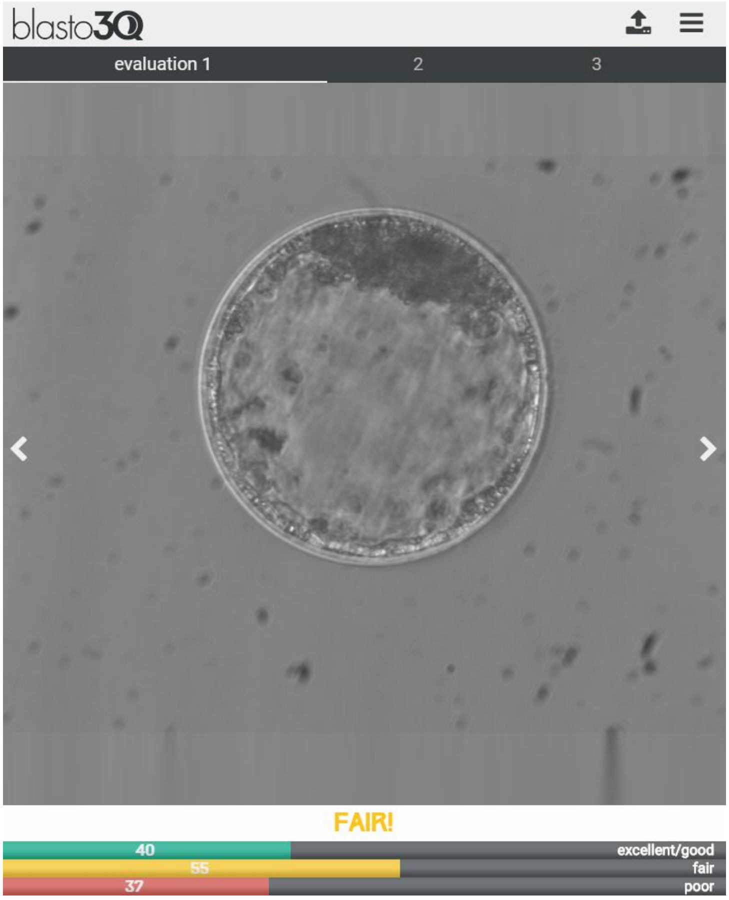

Figure S12. Interface of the Blasto3Q for a mobile smartphone or a PC.

7. References

1. Atherton, T. J. & Kerbyson, D. J. Size invariant circle detection. *Image Vis. Comput.* **17,** 795–803 (1999).

2. Otsu, N. Threshold selection method from gray-level histograms. *IEEE Trans. Syst. Man Cybernet* **9,** 62–66 (1979).
